# Phaeohyphomycosis: A 10-Year Study From a Tertiary Care Centre in South India

**DOI:** 10.7759/cureus.67718

**Published:** 2024-08-25

**Authors:** Rajeswari Kathiah, Saraswathy M P, Sathish Selvakumar, Ranjani Mohan

**Affiliations:** 1 Pathology, All India Institute of Medical Sciences, Madurai, Madurai, IND; 2 Microbiology, ESIC Medical College & PGIMSR, KK nagar, Chennai, IND; 3 Pathology, ESIC Medical College & PGIMSR, KK Nagar, Chennai, IND

**Keywords:** epidemiology, pathology, cutaneous and subcutaneous fungal infections, pigmented fungi., dematiaceous fungi, phaeoid fungi, phaeohyphomycosis

## Abstract

Background

Phaeohyphomycosis, a fungal infection caused by dematiaceous fungi, presents a significant health challenge affecting both immunocompromised and immunocompetent individuals. Despite its clinical importance, phaeohyphomycosis remains underrepresented in epidemiological studies, leading to gaps in our understanding of its prevalence, clinical manifestations, and associated risk factors. This retrospective study conducted in South India aims to address these gaps by examining the incidence, diverse clinical presentations, and other relevant epidemiological aspects of phaeohyphomycosis in patients referred for pathological examination.

Objective

To investigate the epidemiological trends, clinicopathological characteristics, and microbiological spectrum of phaeohyphomycosis in patients at a tertiary care center in South India over 10 years.

Materials and methods

This comprehensive study was conducted at Employees State Insurance Corporation Medical College & Post Graduate Institute of Medical Sciences and Research (ESIC Medical College and PGIMSR), Chennai, embodying a retrospective observational approach. Over a decade, researchers meticulously reviewed cases diagnosed with phaeohyphomycosis. This involved an in-depth analysis of patients' medical records to gather detailed information on presenting symptoms, history of thorn pricks, diabetic status, and other pertinent epidemiological data. Additionally, culture samples were selectively obtained from patients exhibiting abscesses or cystic swellings, followed by a thorough assessment of the culture reports.

Results

In the ten-year study period, a total of 46 cases were identified. Most lesions were solid or cystic and located on extremities, predominantly affecting the digits. Eight cases (17%) had a history of thorn prick injuries, and six cases (13%) were associated with diabetes mellitus. Microscopic examination revealed necrosis, granulomas, varying degrees of inflammatory infiltrates, giant cells, and pigmented fungal hyphae. In some cases, biopsies revealed pseudoepitheliomatous hyperplasia. Among the 19 cases where culture was performed, Alternaria was the most commonly isolated pathogen (42%).

Conclusion

The study brings to light the diagnostic challenges inherent in phaeohyphomycosis cases, which often eluded clinical diagnosis and were only conclusively identified via pathological examinations. While this research was primarily focused on outpatients presenting with minor symptoms, it underscores the potential for more severe clinical presentations in immunocompromised patients. Our findings emphasize the need for increased clinical awareness and the pivotal role of histopathological examination in accurately diagnosing phaeohyphomycosis, particularly in cases with extremity lesions. This study contributes significantly to the understanding of phaeohyphomycosis and advocates for ongoing research to better understand its epidemiology and clinical diversity.

## Introduction

Phaeohyphomycosis represents a complex group of fungal infections characterized by the presence of pigmented (dematiaceous) fungi. These fungi are notable for their distinctive morphological features in tissue, including hyphae, yeast-like cells, or a combination of both. The term "phaeohyphomycosis" was coined to categorize infections caused by these melanin-containing filamentous fungi. Historically uncommon, phaeohyphomycosis has seen a global increase in reported cases in recent years, indicating a rising clinical and epidemiological significance [1,2,3].

Melanin, present in the cell walls of these fungi, is believed to play a crucial role in their pathogenicity, acting as a virulence factor [3]. The clinical spectrum of phaeohyphomycosis is remarkably diverse, affecting both immunocompetent and immunocompromised individuals. However, the clinical manifestations and prognosis differ markedly between these groups. Immunocompromised patients are more likely to develop severe, deep-seated systemic infections, often with a higher mortality risk [4,5].

The management of phaeohyphomycosis varies based on the infection's severity and location. Subcutaneous phaeohyphomycosis typically requires local excision, while more invasive infections necessitate the use of systemic antifungals such as intravenous amphotericin B or azoles, sometimes in conjunction with surgical intervention. Ulcerative skin and soft tissue lesions, particularly those without an associated cyst, can often be managed effectively through debridement [6,7].

Although there exists a plethora of case reports and a few case series on phaeohyphomycosis, comprehensive studies that discuss its epidemiology, and clinical presentation, and correlate these with pathological and microbiological findings are limited. Recognizing this gap in the literature, our study embarked on a retrospective journey to elucidate the epidemiological trends, clinicopathological characteristics, and microbiological spectrum of phaeohyphomycosis, particularly focusing on data from a tertiary care center in South India. This endeavor aims not only to contribute valuable insights into the disease's patterns but also to aid in the development of more effective diagnostic and therapeutic strategies.

## Materials and methods

Study design and approval

This retrospective study was conducted collaboratively by the Department of Pathology and the Department of Microbiology. It received approval from the Institutional Ethics Committee, ensuring adherence to ethical standards in research (IEC number: IEC/ 2022/ 1/ 26).

Case selection

The study encompassed all cases reported with features suggestive of phaeohyphomycosis in biopsy and fine needle aspiration cytology (FNAC) samples received between January 2012 and December 2022. Biopsy samples were preserved in formalin before referral to the Department of Pathology. FNAC was performed for patients with superficial and deep palpable lesions.

Sample collection and processing

In cases presenting with soft and cystic lesions, where aspirates were pus, samples were collected under sterile aseptic conditions and transferred from the Department of Pathology to the Mycology laboratory in the Department of Microbiology for culture.

Diagnostic method

Histopathological examinations of biopsy samples and cytopathological assessments of FNAC cases were conducted using Hematoxylin and Eosin, along with Periodic Acid Schiff stains. In the Mycology laboratory, the samples underwent direct microscopic examination with potassium hydroxide (KOH) wet mount, calcofluor white staining, and fungal culture. The Masson-Fontana stain was employed to demonstrate the presence of melanin, bolstering the diagnosis of phaeohyphomycosis. However, molecular testing, the gold standard for species identification, was not performed due to its unavailability in our institution.

Data collection

Demographic data and relevant clinical details such as lesion site and size, history of thorn prick, and diabetic status were recorded wherever available. Histopathological and cytopathological findings were meticulously documented. Fungal culture reports were obtained from the Department of Microbiology. Additionally, patients' treatment histories were compiled from hospital records.

Statistical analysis

Following comprehensive data collection, statistical analysis was conducted to interpret and summarize the findings, providing a robust foundation for understanding the epidemiological and clinical patterns of phaeohyphomycosis.

## Results

Patient demographics and epidemiology

The study encompassed a total of 46 cases, incorporating referrals for both histopathology and cytopathology. The age distribution ranged from 11 to 70 years, with the majority falling within the 41-50 age group (Table 1). The patient cohort comprised 20 females and 26 males. Unfortunately, data regarding patient occupations, which could be pertinent to understanding phaeohyphomycosis exposure, was not available in the records.

**Table 1:** Age distribution of the patients

Age group	No of patients	% of cases
11 – 20 years	1	2
21 – 30 years	3	7
31 – 40 years	10	22
41 – 50 years	13	28
51 – 60 years	15	32
61 – 70 years	4	9
Total	46	

Clinical history and presentation

Of the cases, 8 (17%) had a history of thorn prick injuries, and 6 (13%) were diagnosed with diabetes mellitus. However, detailed data regarding diabetes control or HbA1c levels were not available for further analysis. All patients exhibited lesions on their extremities, with digits being the most commonly affected site (39%). All patients presented with solitary lesions, and no cases involved multiple lesions. The lesion sizes varied, ranging from 1 to 5 cm in their largest dimension. Notably, 21 cases (46%) presented with cystic swelling or abscesses, while the rest (54%) had solid firm swellings (Table 2).

**Table 2:** Details of patients diagnosed with phaeohyphomycosis PSE: Pseudoepitheliomatous hyperplasia, FBGC: Foreign body giant cells.

Age / Sex	Biopsy or FNAC	Site	Size (Cms)	Other relevant clinical details	Findings other than septate pigmented fungal hyphae	Culture report A / NA
35/F	B	Left index finger	3X2.5	Thorn prick+	Granuloma+ Inflammatory cells+ PSE+	Not done
42/M	B	Right little finger	2X2	-	Granuloma+ Inflammatory cells+	Not done
54/M	B	Right - Calf	4X3	Diabetic	Necrosis+ Inflammatory cells+ PSE+	Not done
58/M	B	Right forearm	2X1.5	-	Necrosis+ Inflammatory cells+	Not done
63/F	C	Right thumb	2X2	-	Necroinflammation+	Alternaria
48/M	C	Left middle finger	2X1	-	Necroinflammation+	Exophiala
47/F	B	Left foot	2X1	-	Granuloma+ Inflammatory cells+	Not done
39/M	B	Right wrist	3X2	Diabetic	Necrosis+ Inflammatory cells+ PSE+	Alternaria
42/M	C	Right knee	4X3	-	Necroinflammation+	Cladophialophora
56/F	B	Left ankle	4X3	Diabetic	Necrosis+ Inflammatory cells+	Not done
39/F	B	Left foot	3X2	-	Granuloma+ Inflammatory cells+	Alternaria
38/F	C	Left index finger	2X1.5	Thorn prick+	Necroinflammation+ FBGC +	Cladophialophora
59/M	B	Right thumb	2X1	-	Necrosis+ Inflammatory cells+	Not done
52/M	B	Left foot	3X2	-	Necrosis+ Inflammatory cells+ PSE+	Not done
50/F	B	Right foot	2X1.5	-	Granuloma+ Inflammatory cells+	Not done
37/M	C	Right thumb	2X1	-	Necroinflammation+	Alternaria
22/M	C	Left foot	2X1.5	-	Necroinflammation+	Exophiala
49/F	B	Left - Calf	3X2	Diabetic	Necrosis+ Inflammatory cells+	Not done
47/M	B	Right little finger	1.5X1	-	Granuloma+ Inflammatory cells+ PSE+	Not done
56/F	B	Right index finger	2.5X1	-	Necrosis+ Inflammatory cells+	Not done
63/F	C	Right palm	2X2	Thorn prick+	Necroinflammation+ FBGC +	Alternaria
29/F	B	Right wrist	4X3	-	Granuloma+ Inflammatory cells+ PSE+	Not done
34/M	C	Right foot	3X2.5	Thorn prick+	Necroinflammation+	Exophiala
59/M	B	Left forearm	4X2.5	-	Granuloma+ Inflammatory cells+ PSE+	Not done
62/M	B	Left palm	3X2.5	-	Granuloma+ Inflammatory cells+ PSE+	Not done
69/M	C	Left foot	3X2	-	Necroinflammation+	Exophiala
60/F	C	Left index finger	2X2	-	Necroinflammation+	Scopulariopsis
54/M	C	Left index finger	1.5X1	-	Necroinflammation+	Not done
51/M	B	Right foot	2.5X2	Thorn prick+	Granuloma+ Inflammatory cells+ PSE+ FBGC +	Not done
46/F	B	Right middle finger	2X2	-	Granuloma+ Inflammatory cells+ PSE+	Not done
42/F	C	Right thumb	1.5X1	-	Necroinflammation+ FBGC +	Not done
59/M	B	Right forearm	2X1.5	-	Granuloma+ Inflammatory cells+ PSE+	Not done
29/M	C	Left wrist	4X3	-	Necroinflammation+	Cladophialophora
33/M	C	Left foot	2X2	Thorn prick+	Necroinflammation+ FBGC +	Alternaria
18/M	B	Right little finger	2X1.5	-	Granuloma+ Inflammatory cells+ PSE+	Not done
43/M	B	Left index finger	1.5X1	-	Granuloma+ Inflammatory cells+ PSE+	Not done
53/F	B	Left palm	2X2	-	Granuloma+ Inflammatory cells+ PSE+	Not done
59/M	C	Left thigh	5X3.5	Diabetic	Necroinflammation+	Alternaria
60/F	C	Left little finger	1.5X1	-	Necroinflammation+	Curvularia
42/M	B	Left foot	2.5X2	-	Granuloma+ Inflammatory cells+ PSE+	Not done
48/F	B	Right knee	3.5X3	-	Necrosis+ Inflammatory cells+ PSE+	Not done
41/M	C	Right thumb	1.5x1	Thorn prick+	Necroinflammation+	Not done
51/F	C	Right foot	2X2	-	Necroinflammation+	Not done
37/F	C	Left little finger	1.5X1	-	Necroinflammation+	Curvularia
33/M	C	Right - Calf	4X3.5	Diabetic	Necroinflammation+	Alternaria
35/F	C	Left foot	2X1.5	Thorn prick+	Necroinflammation+	Scopulariopsis

Pathological findings

**Figure 1:**
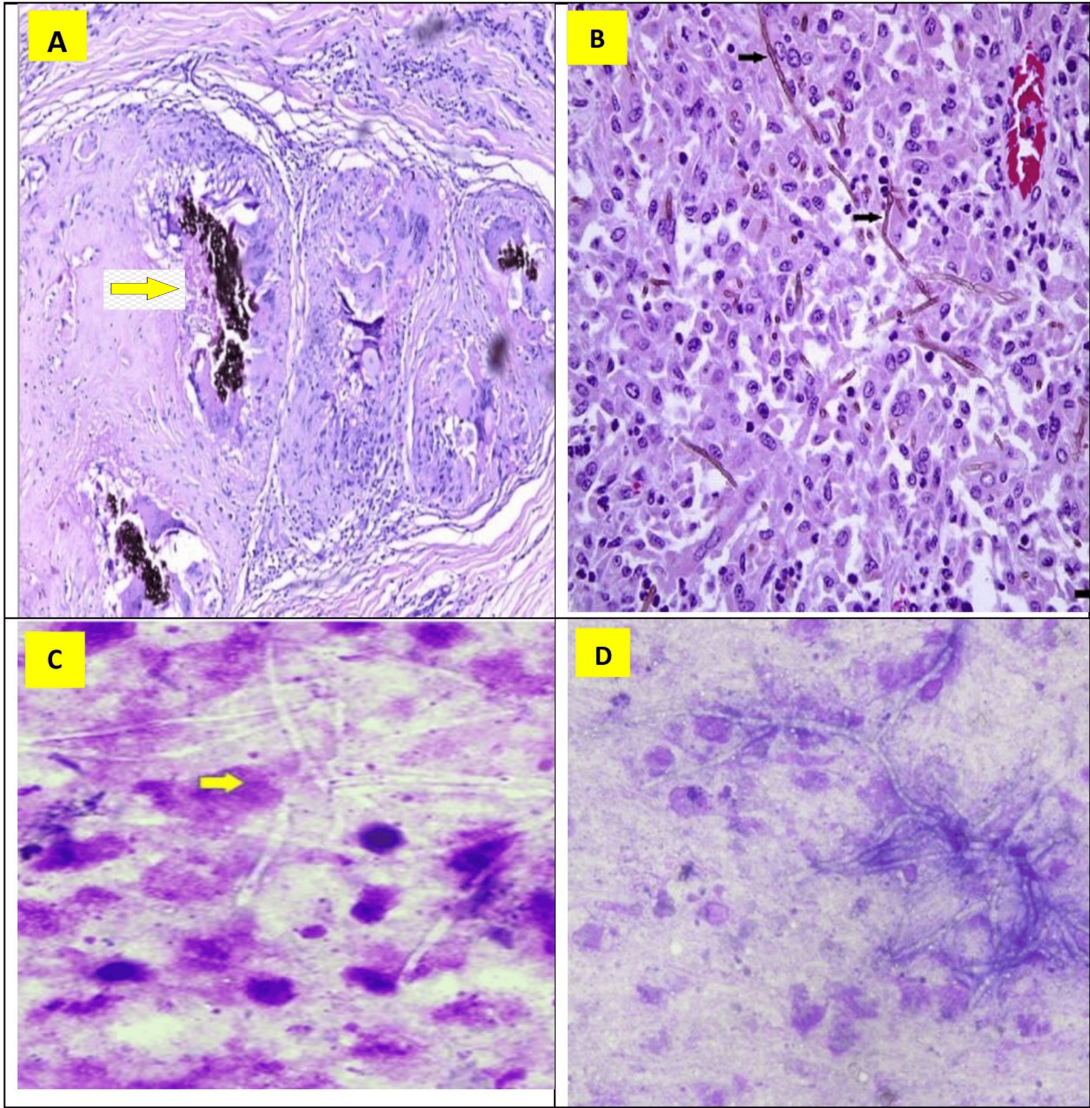
Microscopy - Histopathology and Cytology A&B: Histopathology – photomicrograph showing the presence of pigmented fungal hyphae amidst giant cell reaction, H&E, low power, 100 X. Black arrow points the hyphae. C&D: Cytology – photomicrograph showing the presence of fungal hyphae which are negatively stained and branched, H&E, low power, X100. The yellow arrow points to the hyphae.

The cases presenting with cystic swellings or abscesses underwent FNAC, and sterile material was aspirated for culture and sensitivity testing in the microbiological laboratory. Microscopic examination of biopsies and smears revealed a spectrum of findings, including necrosis, granuloma, inflammatory infiltrate, giant cells, and pigmented fungal hyphae. In 16 out of the 46 cases (35%), the biopsies additionally exhibited pseudoepitheliomatous hyperplasia of the overlying skin. Cytological smears often showed negatively stained and branching fungal hyphae (Figure 1).

Microbiological spectrum

Among the 19 cases that underwent fungal culture, Alternaria emerged as the most common pathogen, accounting for 42% of the isolates. Scopulariopsis and Curvularia, meanwhile, were identified in 11% of cases, representing the least common types (Table 3).

**Table 3:** Causative organisms based on the culture report

Organism	Numbers	% of cases
Alternaria	8	42
Exophiala	4	21
Cladophialophora	3	15
Scopulariopsis	2	11
Curvularia	2	11
Total	19	

Treatment and outcomes

All patients in the study presented with focal lesions. Treatment strategies varied from surgical excision to surgical debridement, without the use of antifungals. Remarkably, all patients responded well to the treatment, with no reported instances of recurrence.

## Discussion

Phaeohyphomycosis is a rare infection caused by dematiaceous fungi, primarily affecting the skin and subcutaneous tissue. The infection results from traumatic inoculation, often through thorn pricks or other injuries. In immunocompromised individuals, phaeohyphomycosis can present with deeper and more severe infections, including systemic involvement [8,9]. The infection generally results from traumatic inoculation of the skin and subcutaneous tissue with contaminated matter. Involvement is typically seen in the upper and lower limbs, particularly on the fingers, wrists, knees, or ankles, with less frequent occurrences on the face, neck, and scalp [10]. Clinically, it often presents as a solitary subcutaneous cyst or abscess, firm to fluctuant, usually sparing the overlying skin [11].

In our study, the most affected age group was 41-50 years, aligning with findings from other studies [11,12]. This suggests that individuals in their most active years are at heightened risk, although no age group is completely immune to infection. We observed a slight male predominance (male-to-female ratio of 1.3:1), which is consistent with most studies but contrasts with some reports indicating a female preponderance [12,13]. This could be attributed to men's increased exposure to outdoor activities, thus elevating their risk.

Diabetes mellitus was identified as a risk factor in 13% of our cases, a finding that varies across different studies [13,14]. Additionally, a history of thorn prick, a significant etiological factor in phaeohyphomycosis, was noted in 17% of our cases, mirroring similar studies [15]. In all patients, the lesions were located on the extremities, especially the digits, consistent with literature reports [16]. Lesion characteristics were predominantly cutaneous or subcutaneous nodules, cysts, or abscesses.

Treatment approaches in our study involved surgical excision or debridement, as phaeohyphomycosis was diagnosed post-pathological examination. Notably, antifungal medications were not administered to any patient. Microscopic examination revealed a spectrum of findings consistent with the literature, including necrosis, granuloma, inflammatory infiltrate, giant cells, and pigmented fungal hyphae. In some cases, biopsies also showed pseudoepitheliomatous hyperplasia of the overlying skin [17].

Masson-Fontana staining, specific for melanin and crucial for confirming dematiaceous fungi, was positive in 17 of the 21 cases referred for microbiological examination. The significance of the Masson-Fontana stain in diagnosing phaeohyphomycosis is well-documented in medical literature, further corroborating the findings of our study.

Limitations

Our study, while providing valuable insights into phaeohyphomycosis, has certain limitations that must be acknowledged:

Sample Bias

The study may inadvertently present a skewed perspective, suggesting that phaeohyphomycosis predominantly affects the skin and subcutaneous tissue in healthy individuals. This bias stems from the fact that all samples and patients included were referred to the Pathology laboratory without prior clinical suspicion of phaeohyphomycosis. Consequently, cases involving inpatients with more severe infections or sepsis, where samples were directly sent to the Department of Microbiology, were not included in our study. This exclusion limits the representation of the full spectrum of phaeohyphomycosis, particularly its manifestation in seriously ill or immunocompromised patients.

Scope of Study

Our retrospective study's scope was restricted to observational analysis, predominantly based on pathological findings. The absence of molecular testing, which is considered the gold standard for identifying fungal species, is a significant limitation. This lack of molecular characterization potentially impacts the accuracy of identifying the fungal species involved and understanding the complete epidemiological and clinical profile of phaeohyphomycosis.

Need for Comprehensive Research

The findings highlight the necessity for larger, more extensive studies that incorporate molecular testing. Such studies would enable a more comprehensive understanding of phaeohyphomycosis, including its varied clinical presentations, epidemiology, and molecular characteristics. Future research should aim to include a broader patient population, encompassing cases with severe systemic involvement, to provide a more holistic view of the disease.

## Conclusions

Phaeohyphomycosis, though uncommon, poses a significant public health concern due to its ability to cause serious disease in both immunocompetent and immunocompromised individuals. Dematiaceous fungi, the causative agents, are widespread in the environment but rarely lead to infection. However, when they do, these infections can manifest in various invasive forms, from deep local infections to pulmonary, cerebral, and disseminated diseases, the latter being particularly fatal. Our study reveals that phaeohyphomycosis may be more prevalent in the community than previously recognized, with immunocompetent individuals often presenting with cutaneous and subcutaneous nodules, cysts, and abscesses.

The study underscores the diagnostic challenge of phaeohyphomycosis, particularly in patients with cutaneous or subcutaneous lesions, even in the absence of common risk factors such as thorn prick injuries or diabetes. Recognizing this condition in differential diagnoses is crucial for timely and accurate treatment, given its potential to cause severe disease across different patient populations.
